# The complete mitochondrial genome of *Anelytra* (*Anelytra*) *hainanensis* (Orthoptera: Tettigoniidae: Conocephalinae)

**DOI:** 10.1128/mra.00187-26

**Published:** 2026-07-27

**Authors:** Nazhen Liu, Xun Bian, Bin Zhang

**Affiliations:** 1 College of Life Sciences & Technology, Inner Mongolia Normal University 71203, Hohhot, China; 2 Key Laboratory of Ecology of Rare and Endangered Species and Environmental Protection (Guangxi Normal University), Ministry of Education 12388, Guilin, China; 3 Key Laboratory of Biodiversity Conservation and Sustainable Utilization for College and University of Inner Mongolia Autonomous Region, Hohhot, China; Rochester Institute of Technology, Rochester, New York, USA

**Keywords:** mitogenome, China, Conocephalinae, *Anelytra (Anelytra) hainanensis *conehead

## Abstract

We present the complete mitochondrial genome of *Anelytra* (*Anelytra*) *hainanensis* Shi, 2015 from Hainan, China. The mitogenome is circular, AT-rich (68.2%), and 15,553 bp in length, comprising 13 protein-coding genes, 2 ribosomal RNA genes, 22 transfer RNA genes, and 1 AT region.

## ANNOUNCEMENT

Orthoptera is a key model for resolving basal insect relationships and investigating the early diversification of winged insects, and Conocephalinae (also referred to as conehead bush crickets) represents a fully wingless group within this order. The species of the genus *Anelytra* are concentrated in the tropical and subtropical zones of Asia (1, 2). *Anelytra* (*Anelytra*) *hainanensis* was first described from Hainan, China (3). Lack of mitogenomic data impedes detailed phylogenetic analyses of *Anelytra*. We therefore assembled and annotated the complete mitogenome of *A. hainanensis* from Hainan to support phylogenomic research on *Conocephalinae*.

The voucher specimen of *A. hainanensis* was collected from Haikou, Hainan, China (19.93°N, 110.21°E) and deposited at Guangxi Normal University. Total genomic DNA was extracted from the hind femur muscle tissues strictly following the instruction manual of the TIANGEN TIANamp Genomic DNA Extraction Kit, and then submitted to Beijing Berry Genomics Co., Ltd. for high-throughput sequencing using an Illumina NovaSeq 6000 sequencer (Illumina Inc.) with the construction of a 150-bp paired-end library. Preprocessing of raw data was performed with fastp v.0.20.0 (4) through adapter and primer trimming, alongside the exclusion of reads with Phred quality <Q5 and reads with a number of N bases >3. A newly generated sequence with a length of 30,830,400 bp was assembled using NOVOPlasty 4.3.3 (5). The Geneious Prime 2025.0.2 was utilized for the annotation of the assembled mitogenome, following the standard and universally accepted start and stop codons based on the invertebrate mitochondrial genetic code (6). The mitochondrial genome map was generated using OGDRAW (7).

**Fig 1:**
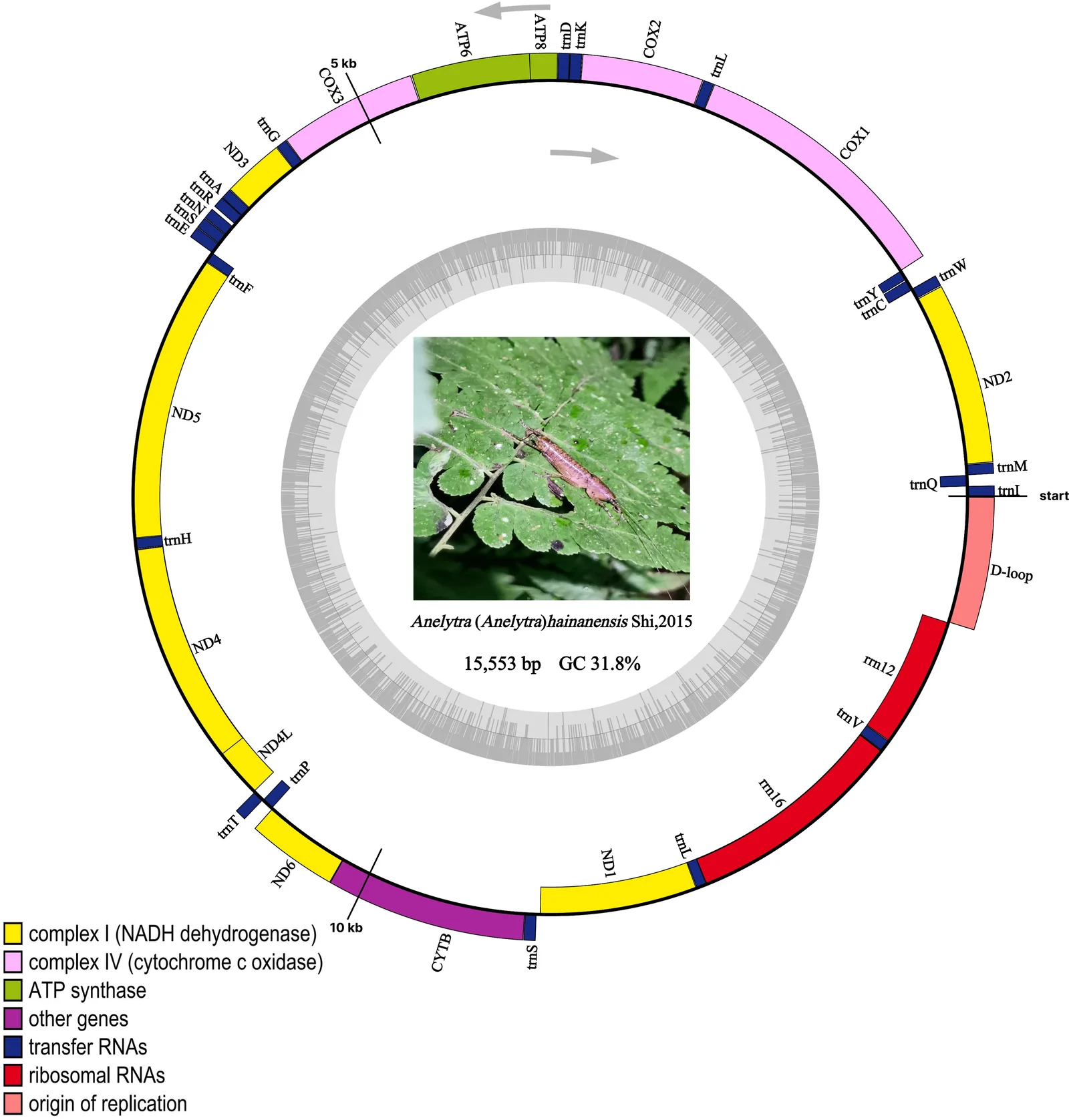
The complete mitochondrial genome map of *Anelytra* (*Anelytra*) *hainanensis* Shi, 2015. The GC content and transcription directions (marked by two arrows) are presented, with the outermost circle indicating the genes. Genes transcribed in the clockwise direction are located internally, and those with counterclockwise transcription are positioned externally. Different gene groups are distinguished by color coding, as specified in the legend.

The complete mitogenome of *Anelytra* (*Anelytra*) *hainanensis* Shi, 2015 (PX872203.1) is a cyclic double-stranded structure with a length of 15,553 bp (Fig. 1), and the GC content is 31.8%. The mitogenome shows a strong AT bias, which is typical of orthopteran insects. Its base composition is highly similar to that of *Anelytra eunigrifrons* (33.4%) (8), indicating consistent base compositional characteristics between the two *Anelytra* species. The mitogenome consists of one control region and 37 genes (most insect mitochondrial genomes contain the standard set of 37 genes), including 13 protein-coding genes (PCGs), 22 transfer RNA genes (tRNAs), and 2 ribosomal RNA genes (16S rRNA and 12S rRNA). Among the 13 PCGs, all use ATN as the start codon except *COX1* (CCG) and *ND1* (GTG) (9) (Table 1). All other PCGs use TAA as the stop codon except *ND1* (TAG), *ND3* (TA), and *COX1*, *COX2*, *COX3*, *ND4*, *ND5*, *CYTB* (T). As a prevalent feature in animal mitochondrial genomes, the incomplete T stop codon is completely formed by the addition of 3′ A residues to mRNA (10, 11). The gene order is identical to the ancestral gene arrangement of Orthoptera, and no gene rearrangement was observed, suggesting that the mitogenome of *Anelytra* is evolutionarily conserved. The newly sequenced mitogenome of *A. hainanensis* enriches the genomic resources of Conocephalinae. It contributes to resolving phylogenetic relationships and species delimitation within this subfamily, and also serves as fundamental data for investigating mitogenomic evolution in orthopteran insects. Furthermore, this mitogenome provides reliable molecular evidence for the morphological taxonomy of *Anelytra* and aids in the discrimination of morphologically allied species.

**TABLE 1:** Gene composition and annotation of *Anelytra* (*Anelytra*) *hainanensis* Shi, 2015

Gene	Type	Minimum nucleotide position	Maximum nucleotide position	Length	Start codon	Stop codon	Direction	Protein length
*tRNA-Ile*	tRNA	1	64	64	–^*a*^	–	Forward	–
*tRNA-Gln*	tRNA	62	130	69	–	–	Reverse	–
*tRNA-Met*	tRNA	129	192	64	–	–	Forward	–
*ND2*	CDS	194	1225	1,032	ATC	TAA	Forward	343
*tRNA-Trp*	tRNA	1228	1293	66	–	–	Forward	–
*tRNA-Cys*	tRNA	1286	1353	68	–	–	Reverse	–
*tRNA-Tyr*	tRNA	1357	1420	64	–	–^*a*^	Reverse	–
*COX1*	CDS	1419	2952	1,534	CCG	T	Forward	511
*tRNA-Leu*	tRNA	2953	3016	64	–	–	Forward	–
*COX2*	CDS	3018	3708	691	ATG	T	Forward	230
*tRNA-Lys*	tRNA	3709	3778	70	–	–	Forward	–
*tRNA-Asp*	tRNA	3778	3845	68	–	–	Forward	–
*ATP8*	CDS	3846	4007	162	ATT	TAA	Forward	53
*ATP6*	CDS	4001	4675	675	ATG	TAA	Forward	224
*COX3*	CDS	4679	5465	787	ATG	T	Forward	262
*tRNA-Gly*	tRNA	5466	5530	65	–	–	Forward	–
*ND3*	CDS	5531	5883	353	ATC	TA	Forward	117
*tRNA-Ala*	tRNA	5884	5946	63	–	–	Forward	–
*tRNA-Arg*	tRNA	5946	6011	66	–	–	Forward	–
*tRNA-Asn*	tRNA	6029	6094	66	–	–	Forward	–
*tRNA-Ser*	tRNA	6095	6161	67	–	–	Forward	–
*tRNA-Glu*	tRNA	6162	6228	67	–	–	Forward	–
*tRNA-Phe*	tRNA	6227	6290	64	–	–	Reverse	–
*ND5*	CDS	6291	8022	1,732	ATT	T	Reverse	577
*tRNA-His*	tRNA	8023	8087	65	–	–	Reverse	–
*ND4*	CDS	8088	9426	1,339	ATG	T	Reverse	446
*ND4L*	CDS	9420	9716	297	ATG	TAA	Reverse	98
*tRNA-Thr*	tRNA	9719	9787	69	–	–	Forward	–
*tRNA-Pro*	tRNA	9787	9860	74	–	–	Reverse	–
*ND6*	CDS	9862	10380	519	ATA	TAA	Forward	172
*CYTB*	CDS	10380	11514	1,135	ATG	T	Forward	378
*tRNA-Ser*	tRNA	11515	11583	69	–	–	Forward	–
*ND1*	CDS	11603	12550	936	GTG	TAG	Reverse	311
*tRNA-Leu*	tRNA	12551	12615	65	–	–	Reverse	–
*16S rRNA*	rRNA	12615	13935	1,321	–	–	Reverse	–
*tRNA-Val*	tRNA	13935	14005	71	–	–	Reverse	–
*12S rRNA*	rRNA	14006	14797	792	–	–	Reverse	–

^*a*^ –, not applicable.

## Data Availability

The complete mitochondrial genome sequence of *Anelytra* (*Anelytra*) *hainanensis* Shi, 2015 is available in GenBank under accession number PX872203.1. The associated BioProject, SRA, and BioSample numbers are PRJNA1404717, SRR36983366, and SAMN54723044, respectively.
